# Increased runs of homozygosity in the autosomal genome of Brazilian individuals with neurodevelopmental delay/intellectual disability and/or multiple congenital anomalies investigated by chromosomal microarray analysis

**DOI:** 10.1590/1678-4685-GMB-2020-0480

**Published:** 2022-02-28

**Authors:** Gabriela Roldão Correia-Costa, Ilária Cristina Sgardioli, Ana Paula dos Santos, Tânia Kawasaki de Araujo, Rodrigo Secolin, Iscia Lopes-Cendes, Vera Lúcia Gil-da-Silva-Lopes, Társis Paiva Vieira

**Affiliations:** 1 Universidade de Campinas, Faculdade de Ciências Médicas, Departamento de Medicina Translacional, Campinas, SP, Brazil.

**Keywords:** Runs of homozygosity, chromosomal microarray analysis, identity by descent, uniparental disomy

## Abstract

Runs of homozygosity (ROH) in the human genome may be clinically relevant. The aim of this study was to report the frequency of increased ROH of the autosomal genome in individuals with neurodevelopmental delay/intellectual disability and/or multiple congenital anomalies, and to compare these data with a control group. Data consisted of calls of homozygosity from 265 patients and 289 controls. In total, 7.2% (19/265) of the patients showed multiple ROH exceeding 1% of autosomal genome, compared to 1.4% (4/289) in the control group (p=0.0006). Homozygosity ranged from 1.38% to 22.12% among patients, and from 1.53 to 2.40% in the control group. In turn, 1.9% (5/265) of patients presented ROH ≥10Mb in a single chromosome, compared to 0.3% (1/289) of individuals from the control group (p=0.0801). By excluding cases with reported consanguineous parents (15/24), the frequency of increased ROH was 3.4% (9/250) among patients and 1.7% (5/289) in the control group, considering multiple ROH exceeding 1% of the autosome genome and ROH ≥10Mb in a single chromosome together, although not statistically significant (p=0.1873). These results reinforce the importance of investigating ROH, which with complementary diagnostic tests can improve the diagnostic yield for patients with such conditions.

## Introduction

Chromosomal microarray analysis (CMA) has been established as the first-tier diagnostic test for patients with neurodevelopmental disabilities and/or congenital anomalies, offering a diagnostic yield close to 20% for pathogenic copy number variations (CNVs) (Miller et al., 2010; Vermeesch et al., 2012). Besides detecting CNVs, CMA platforms that incorporate single nucleotide polymorphism (SNP) probes also enable the detection of runs of homozygosity (ROH) throughout the genome, that occurs mainly by two mechanisms: (1) identity-by-descent, especially when there is parental consanguinity, and (2) uniparental disomy (UPD), mostly due to a trisomic rescue (Kearney et al., 2011; Wang et al., 2015). Although in most cases with increased ROH detected by CMA, the presence of such regions does not allow a diagnostic conclusion, it does assist in the diagnostic investigation, as the occurrence of these regions significantly increases the risk for rare monogenic diseases with recessive inheritance (Sasaki et al., 2013; Sund et al., 2013; Wang *et al*., 2015; Alabdullatif et al., 2017; Chantot-Bastaraud et al., 2017).

As demonstrated by previous studies investigating ROH prevalence in different populations, the occurrence of these regions seems to be underestimated in the human genome. The frequency and size of these ROH, as well as the genetic conditions related to its occurrence, varies widely from population to population, carrying very important demographic and cultural traits (Gibson et al., 2006; McQuillan et al., 2008; Nothnagel et al., 2010; Bruno et al., 2011; Chaves et al., 2019). Despite the high genetic admixture in the Brazilian population, the country still has some areas with isolated populations, which contributes to the high prevalence of consanguineous marriages and to the occurrence of rare recessive conditions. However, data on these isolated groups and their genetic traits are still scarce in the literature, especially due to the high costs of genomic analyses in countries such as Brazil (Cardoso et al., 2019).

In individuals born of consanguineous parents, the amount of ROH is directly proportional to the level of parental relatedness, ranging from approximately 1% in children born from fifth-degree related parents to nearly 25% in those born from first-degree related parents (Sund et al., 2013). In these cases, genomic analysis shows multiples ROH in different genomic regions, which are identical-by-descent. Conversely, one or more ROH in a single chromosome can be a hallmark of UPD, which can be either whole-chromosome or segmental UPD (Kearney et al., 2011). The aim of this study was to report the frequency of single ROH ≥10Mb or multiple ROH exceeding 1% of the autosomal genome in individuals with neurodevelopmental delay (NDD)/intellectual disability (ID) and/or multiple congenital anomalies (MCA) previously investigated by CMA, as well as to compare these data with a control group from the Brazilian population.

## Subjects and Methods

### Sample

This study was approved by the Research Ethics Committee of the University of Campinas (CAAE number: 02179518.4.0000.5404). The study sample included 265 individuals, most of who were pediatric patients, referred for CMA between 2010 and 2018 in the Laboratory of Human Cytogenetics and Cytogenomics at the School of Medical Sciences of Unicamp; the main clinical indications for CMA were NDD/ID, and/or MCA. The control sample included 289 individuals from the Brazilian general population - 130 from the control group of the Laboratory of Human Cytogenetics and Cytogenomics and 159 from the Brazilian Initiative on Precision Medicine (BipMed) repository (Rocha et al., 2020).

### Chromosomal microarray analysis

Both patients and controls were tested using CMA chips from Affymetrix^®^ - Thermo Fisher Scientific Inc. (Life Technologies, Carlsbad, CA, USA), among which 169 patients and 110 controls were tested with the CytoScan™ HD; 79 patients with the CytoScan™ 750K chip; and 17 patients and 179 controls with the *Genome-Wide Human* SNP *Array* 6.0™ chip. The data were analyzed using the Affymetrix Chromosome Analysis Suite (ChAS - Santa Clara, CA, USA) version 4.0. 

For the ROH calling, a minimal number of 500 probes and a minimal size of 1.500 kb were considered. Using the allele-peak charts of the Chromosome Analysis Suite (ChAS), each region of homozygosity meeting this criterion was visually checked. For the purpose of this study, which followed the European Guideline for Constitutional Cytogenomic Analysis (Silva et al., 2019) to detect clinically-relevant ROH, only ROH ≥10Mb in a single chromosome and multiple ROH whose sum exceeded 1% of the autosomal genome were considered - even though pathogenic variants can be encompassed in ROH smaller than 10Mb or not achieving 1% of the autosome genome. 

Each platform used has different numbers of SNP probes (according to the manufacturer, the SNP array 6.0 chip includes about 906,600 probes; the CytoScan HD chip about 743,304 probes; and the the CytoScan 750K chip about 200,436 probes) and, consequently, different coverage densities for ROH detection. Since this analysis was concerned with detecting only long stretches of ROH (≥10Mb, or ≥1% of the autosome genome), we verified no impact with the use of different chips.

The percentage of homozygosity (*%*_*roh auto*_ ) in the genome of each individual was calculated as suggested by Kearney et al. (2011): by dividing the sum of all homozygous regions in autosomes (Σ_roh auto_), by total autosomal length (3020Mb for *GRCh37 - hg19*) and multiplying the result by 100. The presence of imprinted genes within the ROH on single chromosomes, which suggests UPD, was verified using the Geneimprint database (http://www.geneimprint.com/).

Statistical analysis was performed using the Pearson’s Chi-square test or Fisher’s Exact test. Each variant (single ROH ≥10Mb, multiple ROH exceeding 1% and multiple ROH lower than 1%) was calculated independently, always in comparison with the control group. Calculations were performed in the 2016 Microsoft Excel (version 1.0) and p < 0.05 was considered statistically significant.

## Results

**Figure 1 -:**
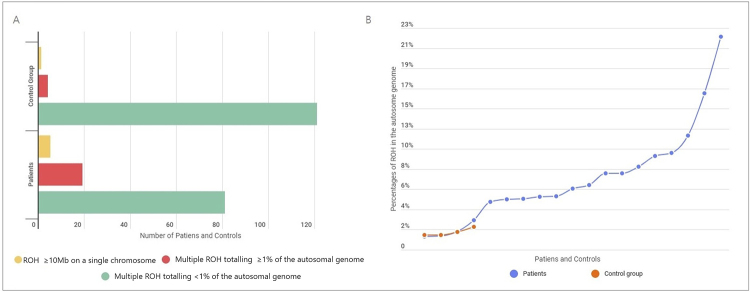
(A) Number of patients and controls with homozygous regions in the autosomal genome. Patients show a higher prevalence of clinically relevant ROH (single ≥10Mb and multiple ≥1%), as expected (5/265 versus 1/289 and 19/265 versus 4/289, respectively). However, multiple ROH, that do not exceed 1% of the autosomal genome and that unlikely have clinical relevance, are more frequent in the control group (81/265 versus 121/289); (B) Values referring to percentages of homozygosity in the autosomal genome of 19 patients and four controls whose ROH sum exceeds 1%. The observed ROH ≥10Mb on a chromosome and multiple ROH totaling ≥ 1% of the genome should be considered with potential clinical relevance.

Multiple ROH exceeding 1% of the total autosomal genome occurred in 19 (7.2%) of the 265 individuals with NDD/ID and/or MCA and in four of the 289 individuals from the control group (1.4%) (p=0.0006). In turn, five patients (1.9%) and one individual from the control group (0.3%) showed ROH ≥10Mb in a single chromosome (p=0.0801) (Figure 1A). Among these, 16 patients and three controls were tested with the Cytoscan HD chip, seven patients with the Cytoscan 750K chip, and one patient and two controls with the SNP array 6.0 chip. We found no pathogenic or likely-pathogenic CNV among the 24 patients with increased ROH.

Moreover, 81 patients (30.6%) and 121 controls (41.8%) presented multiple ROH that, when summed, did not exceed 1% of the autosomal genome (p=0.0058) (Figure 1A). We found no ROH in the autosomal genome of 160 patients (60.3%) and 163 controls (56.5%). Considering both multiple ROH exceeding 1% of the total autosomal genome and ROH ≥10Mb in a single chromosome, 24 patients (9%) and five controls (1.7%) showed increased ROH, demonstrating that such regions are more frequent among patients (p=0.0001). 

The percentage of homozygosity ranged from 1.38% to 22.12% among the 19 patients with multiple ROH exceeding 1% of the autosomal genome and, from 1.53 to 2.40% in the control group (Figure 1B). Given that 15/19 individuals were known to be born from consanguineous parents, such a finding was already expected. Consanguinity was not reported by the families in four cases, with homozygosity ranging from 1.38% to 10.13%. No information on parental relatedness for the control group was available.

**Figure 2 -:**
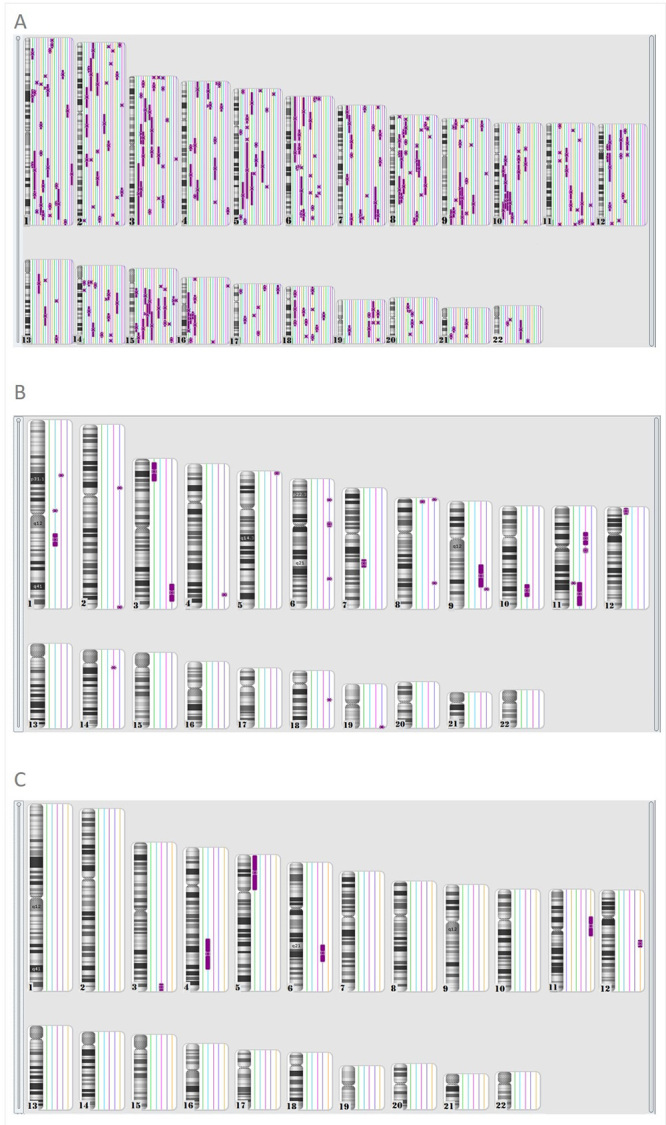
Karyoview from Affymetrix® Chromosome Analysis Suite (ChAS) Software demonstrating: (A) all multiple ROH exceeding 1% of the autosomal genome of the 19 patients; (B) all multiple ROH exceeding 1% of the autosomal genome of the control group; and (C) ROH ≥10Mb in a single chromosome, in patients (chromosomes 3, 4, 5, 6 and 12) and controls (chromosome 11).

Regarding genomic location of increased ROH, partial overlapping between different blocks of homozygosity in the 19 patients with multiple ROH exceeding 1% of the autosomal genome was observed (Figure 2A). As none of these patients are related and more than 5% of ROH were detected in 14 individuals, five of which with more than 10% of ROH, such overlapping might have occurred by chance. However, potential mechanisms such as low recombinant rates in these genomic regions cannot be excluded. We found no recurrent region with significant increased homozygosity in the four individuals with multiple ROH exceeding 1% of the autosomal genome from the control group (Figure 2B). Likewise, neither patients nor controls presented recurrent regions with homozygosity ≥10Mb in length (Figure 2C), as well as no imprinted genes in these regions. 

This study focused on runs of homozygosity in autosome chromosomes. However, 11 out of the 19 patients with multiple ROH were female and three of them presented at least one ROH in the X chromosome. Moreover, the four individuals with multiple ROH from the control group were female, two of whom presented one single ROH in the X chromosome.

Table 1 describes the reported level of relatedness of the study sample, as well as the expected (theoretical and admitted) and detected percentages of homozygosity. Considering the error rate proposed by Sund et al (2013), we found that the percentages of ROH detected were compatible with the level of relatedness reported by families for 11 of 15 cases (Table 1). Among the four cases in which such a correspondence was not verified, two (P009 and P011) were from small towns in the Southeast and Northeast regions of Brazil (both with less than 35,000 inhabitants) and the other two (P012 and P013) had multiple consanguineous marriages in previous generations of the family. 

**Table 1 -:** Theoretical, admitted and detected percentages of homozygosis for each patient.

ID	Reported relatedness	Percentages of homozygosity:		
		Theoretical	Admitted^1^	Found
P001	Uncle/niece	12.5%	9.7 - 15.3%	16.24%
P002	1st cousins	6.25%	4.6 - 8.3%	5.28%
P003	1st cousins	6.25%	4.6 - 8.3%	6.34%
P004	1st cousins	6.25%	4.6 - 8.3%	6.74%
P005	1st cousins	6.25%	4.6 - 8.3%	7.92%
P006	1st cousins	6.25%	4.6 - 8.3%	8.64%
P007	1st cousins	6.25%	4.6 - 8.3%	5.23%
P008	1st cousins	6.25%	4.6 - 8.3%	5.56%
P009	1st cousins	6.25%	4.6 - 8.3%	9.73%
P010	1st cousins once removed	3.125%	2.6 - 4.2%	1.88%
P011	1st cousins once removed	3.125%	2.6 - 4.2%	4.98%
P012	1st cousins once removed	3.125%	2.6 - 4.2%	22.12%
P013	2nd cousins	1.5625%	0.5 - 1.6%	11.86%
P014	Unknow related degree^2^	-	-	1.44%
P015	Unknow related degree^2^	-	-	5.51%
P016	Not reported	-	-	1.38%
P017	Not reported	-	-	3.06%
P018	Not reported	-	-	7.92%
P019	Not reported	-	-	10.05%

^1^Percentages of homozygosity admitted, according to Sund et al. (2013). Percentages of homozygosity intermediate to these values should be related to the degree of kinship to which the value is closest. E.g.: a percentage of 16.24% is closer to 15.3% (borderline to 2th degree) than to 21.3% (borderline to 1st degree). Therefore, it is considered relationship in 2nd degree.

^2^Parents reported consanguinity, but do not know the relatedness degree.

Regarding the four patients who presented multiple ROH exceeding 1% and no report of consanguinity, the percentage of homozygosity ranged from 1.05% to 10.13%, indicating different degrees of identity-by-descent. Among these, two (P016 and P017) were from small towns in the Northeast region of Brazil, with less than 26,000 inhabitants, which can be considered regions with more probable increased inbreeding. The other cases (P018 and P019) presented homozygosity of 7.92% and 10.05% and were probably born from related parents, who did not wish to report this information for unknown reasons. 

No consanguineous union was reported among individuals with ROH ≥10Mb in a single chromosome (Table 2), whose length ranged from 10.077Mb to 46.269Mb. The ROH was located in interstitial chromosome regions in four cases, and in a terminal chromosome region in only one individual (P024).

**Table 2 -:** ROH≥10Mb detected on single chromosomes in five patients and one control individual.

ID	Chromosome	Genomic position	Length (pb)	MIM genes associated with recessive diseases
P021^1^	12	arr[GRCh37] 12q14.3q21.2(66001610_76078947) hmz	10,077,337	*GRIP1; IFNG; NUP107; MDM2.*
P022^1^	3	arr[GRCh37] 3q27.3q29(187631077_197851260) hmz	10,211,100	*P3H2; CLDN16; CLDN1; OPA1; TFRC; CEP19; TCTEX1D2; RUBCN; RNF168; PCYT1A; NRROS.*
P023^1^	6	arr[GRCh37] 6q21q23.2(109263688_132108398) hmz	22,844,773	*ARMC2; ZBTB24; FIG4; TRAF3IP2; CCN6;TSPYL1; RFX6; NUS1; MCM9; GJA1; TRDN; LAMA2; ARG1; MED23.*
P022^1^	4	arr[GRCh37] 4q27q32.2(121450632_162682338) hmz	41,232,833	*PRDM5; EXOSC9; BBS7; KIAA1109; IL21; BBS12; SPATA5; FAT4; INTU; PLK4; MFSD8; RAB33B; GAB1; SLC10A7; TTC29; MMAA; LRBA; MAB21L2; GATB; TRIM2; FGB; FGA; FGG; LRAT; TDO2; GUCY1A3; GLRB; ETFDH.*
P024^1^	5	arr[GRCh37] 5p15.33p11(113576_46383335) hmz	46,269,772	*SDHA; SLC9A3; SLC6A19; TRIP13; SLC6A3; TERT; NDUFS6; NSUN2; MTRR; CCT5; OTULIN; FAM134B; SLC45A2; TARS1; AMACR; DNAJC21; NADK2; AGXT2; NUP155; CPLANE1; IL7R; SPEF2; LIFR; FYB1; NNT; GHR; OXCT1.*
BMS09^2^	11	arr[GRCh37] 11p13q12.3(31000001_63400000) hmz	26,292,157	*PDHX; CD59; RAG1; RAG2; EXT2; ALX4; PEX16; ZNF408; F2; DDB2; MADD; MYBPC3; SLC39A13; RAPSN; NDUFS3; SLC35C1; CREB3L1; LRP4; NUP160; C1NH; CLP1; TMX2; CBLIF; ZP1; TKFC; TMEM138; TMEM216; ROM1; B3GAT3 ; BSCL2; UQCC3.*

¹Patients;

²Individual from control group.

## Discussion

Increased ROH in the human genome is considered an important finding for providing clues about ancestral homozygous alleles, consanguinity, and uniparental disomy. Moreover, verifying the occurrence of increased ROH in the autosomal genome of individuals presenting ID and/or MCA, may support in the diagnostic investigation by indicating candidate genes in the search for genes related to a recessive disorder (Sund et al., 2013; Wang et al., 2015). Previous studies reported increased ROH in 1.8% to 12% of the samples (Bruno et al., 2011; Sund *et al.,* 2013; Wang *et al*., 2015; Alabdullatif et al., 2017; Chaves et al., 2019; Ali et al., 2020) however, most of these studies did not compare the sample with a control group.

The results of the present study demonstrated that the occurrence of ROH in the group of patients is higher when compared to the control group. As these disabilities are more frequent among children born from consanguineous families (Hu et al., 2019) and considering that 15 families in this group reported parental relatedness, such a prevalence was already expected. Although not statistically significant (p=0.1873 - Fisher’s Exact test), this higher prevalence of ROH among patients remains even after excluding cases with reported consanguinity, with 9/250 (3.4%) among patients and 5/289 (1.7%) in the control group. 

Regarding multiple ROH exceeding 1% of the total autosomal genome, its proportion was significantly higher in the patients group (p=0.0006). Also, the percentage of ROH was higher among patients (1.38% to 22.12%) than among control individuals (1.53 to 2.40%). The percentage of patients with multiple ROH that did not exceed 1% of the autosomal genome was 30.6%, while that of controls was 41.8% (p=0.0058). Corroborating current guidelines for reporting ROH in CMA results, these findings reinforce the idea that, contrary to ROH whose sum exceeds 1% of the autosomal genome, those whose sum remains below 1% are less likely to be clinically relevant (Silva et al., 2019).

Five patients and one control presented ROH ≥10Mb in a single chromosome, all of which were suggestive of segmental UPD or a distant common ancestor, and none suggestive of whole chromosome UPD. This result may be due to two reasons. Firstly, UPDs of chromosome 11 (Beckwith-Wiedemann and Russell-Silver syndromes) and 15 (Angelman and Prader-Willi syndromes) - the most common syndromes caused by UPD of entire chromosomes - present characteristic phenotypes, being often referred to specific diagnostic tests rather than to CMA, so that cases might have been excluded from this sample. Secondly, because entire-chromosome UPD is a very rare event and both of our samples (patients and controls) were small, this result might have been by chance. However, Nakka *et al.* (2019) reported that uniparental disomy in the general population may be about 1.75 times greater than estimated. Only in one of the cases with ROH in a single chromosome, it occurred in the terminal part of a chromosome arm, thus being more likely to be a segmental UPD. The other cases, with ROH in interstitial chromosome regions, are more likely to be due to a distant common ancestor (Kearney et al., 2011).

The higher prevalence of ROH among patients allows us to infer that these regions may be clinically relevant and that the occurrence of homozygous variants in recessive inheritance genes, mapped within the ROH, can justify the phenotypes in this group of patients. Further investigation, using homozygosity mapping in affected sib pairs, a candidate gene approach, or whole exome sequencing are required to achieve a diagnostic conclusion. The combined analyses of ROH detected by CMA and sequencing methods can increase the diagnostic yield of disorders with recessive inheritance (Sund et al., 2013; Alabdullatif et al., 2017; Prasad et al., 2018).

In a study with 430 Brazilian individuals with neurodevelopmental disorders, Chaves et al. (2019) found 95% of these individuals to have at least one ROH > 3 Mb in length in the autosomal genome, 2.6% of which were suggestive of UPD. Moreover, 8.5% of the cases presented multiple ROH exceeding 1% of the genome, which are more likely to have a clinical impact. The authors also considered ROH present in more than 5% of the patients as common ROH. 

To the best of our knowledge, this is the first study to compare the frequency and percentage of ROH among Brazilian individuals with ID/NDD and/or MCA and a control group. In conclusion, this study showed a higher proportion of clinically relevant ROH among patients with such conditions compared to healthy controls, reinforcing the importance of analyzing and reporting ROH in the autosomal genome of individuals referred for CMA.
